# Expanding the Clinical and Molecular Spectrum of Cartilage-Hair Hypoplasia in the Mexican Population

**DOI:** 10.3390/ijms27198815

**Published:** 2026-10-02

**Authors:** Kiabeth Robles-Espinoza, Eduardo Esparza-García, Jorge Román Corona-Rivera, Mariana Pérez-Coria, Perla Graciela Rodríguez-Gutiérrez, Juan Ramón González García, María Teresa Magaña-Torres

**Affiliations:** 1 División de Genética, Centro de Investigación Biomédica de Occidente, Instituto Mexicano del Seguro Social, Sierra Mojada 800, Independencia Oriente, Guadalajara 44340, Jalisco, Mexico; kiabethre@gmail.com (K.R.-E.);; 2 Doctorado en Genética Humana, Centro Universitario de Ciencias de la Salud, University of Guadalajara (UdeG), Sierra Mojada 950, Independencia Oriente, Guadalajara 44340, Jalisco, Mexico; 3 UMAE Hospital de Pediatría del Centro Médico Nacional de Occidente, Instituto Mexicano del Seguro Social, Av. Belisario Domínguez 735, La Perla, Guadalajara 44360, Jalisco, Mexico; 4 Center for Registry and Research in Congenital Anomalies (CRIAC), Service of Genetics and Cytogenetics Unit—‘Dr. Enrique Corona-Rivera’ Institute of Human Genetics, ‘Dr. Juan I. Menchaca’ Civil Hospital of Guadalajara (HCG), Guadalajara 44340, Jalisco, Mexico; 5 Health Sciences University Center (CRIAC HCG—UdeG), University of Guadalajara (UdeG), Guadalajara 44340, Jalisco, Mexico

**Keywords:** cartilage-hair hypoplasia, *RMRP*, skeletal dysplasia, novel promoter deletion n.-39_-19del

## Abstract

Cartilage-hair hypoplasia (CHH) is a rare autosomal recessive skeletal dysplasia caused by variants in *RMRP* (RNA component of mitochondrial RNA processing endoribonuclease), which encodes a non-coding RNA. Despite its well-established clinical and molecular spectrum in some populations, data from Mexico remain limited. We report eight patients from seven unrelated families with CHH. Clinical and radiographic findings were assessed, and *RMRP* was analyzed by polymerase chain reaction and Sanger sequencing. All patients exhibited disproportionate short stature; prenatal long-bone shortening and short stature at birth occurred in 50%. Radiographs showed widened long bones and metaphyseal dysplasia in all patients. Extraskeletal manifestations were observed in four patients, each with a distinct feature: immunodeficiency, anemia, Hirschsprung disease, or lymphopenia. *RMRP* variants were identified in all patients, comprising two homozygous and six compound heterozygous genotypes. Eight variants were detected: three pathogenic (n.6C>T, n.147G>A, and n.72A>G), two likely pathogenic (n.127C>T and n.125C>T), two variants of uncertain significance (n.128G>C and n.215C>T), and a novel promoter deletion (n.-39_-19del) encompassing the *RMRP* TATA box, classified as likely pathogenic according to the ClinGen *RMRP* variant interpretation specifications (GN088, Version 1.3). This study expands the clinical and molecular spectrum of CHH in the Mexican population and provides additional evidence for *RMRP* variant interpretation.

## 1. Introduction

Cartilage-hair hypoplasia (CHH; OMIM #250250) is an autosomal recessive chondrodysplasia with marked variation in prevalence across populations. Its incidence is approximately 1 in 1300 live births in the Amish population and 1 in 23,000 live births in Finland, whereas the disorder is rare in most other populations [1,2]. CHH is characterized by disproportionate short stature and metaphyseal abnormalities, with variable involvement of the epiphyses of the long bones. Skeletal manifestations include ligamentous laxity, varus deformity, and lumbar lordosis [3].

CHH is a multisystem disorder with a broad and variable clinical spectrum. Historically, biallelic *RMRP* (RNA component of mitochondrial RNA processing endoribonuclease) variants were associated with distinct clinical phenotypes: anauxetic dysplasia, cartilage-hair hypoplasia, and metaphyseal dysplasia without hypotrichosis. These phenotypes are now recognized as part of a broader spectrum of *RMRP*-related disorders, ranging from severe skeletal dysplasia to milder skeletal involvement and including immunodeficiency, anemia, Hirschsprung disease, and increased susceptibility to malignancies [3,4,5]. Diagnosis is suspected based on characteristic clinical and radiographic findings and confirmed by molecular genetic testing [3].

CHH results from disease-causing variants in the *RMRP* gene, located on chromosomal band 9p13.3. Unlike protein-coding genes, *RMRP* is transcribed by RNA polymerase III and encodes a highly conserved non-coding RNA of approximately 269 nucleotides that is an essential structural and functional component of the RNase MRP complex [6,7]. RNase MRP participates in several RNA-processing pathways, including precursor rRNA cleavage required for 5.8S rRNA maturation and ribosome biogenesis, mitochondrial DNA replication, and processing of specific mRNAs involved in cell-cycle regulation [7,8]. In addition, RMRP-derived small RNAs may regulate genes involved in skeletal development, hematopoiesis, and cellular differentiation, potentially contributing to the multisystemic manifestations of CHH [8].

The molecular spectrum of *RMRP* variants is highly heterogeneous, with at least 72 pathogenic and 44 likely pathogenic variants reported to date, including single-nucleotide substitutions, insertions, deletions, and duplications [9,10]. Alterations in the transcribed region may disrupt RNA structure, stability, or RNase MRP function, whereas promoter alterations may impair transcription and reduce RMRP transcript abundance [9]. The common Finnish founder allele n.72A>G (historically designated n.70A>G) has been identified in individuals from diverse populations worldwide [11]. Clinically, the spectrum ranges from relatively mild presentations characterized by short stature and metaphyseal abnormalities to severe forms within the CHH–anauxetic dysplasia spectrum [12]. However, the relationship between *RMRP* alterations and clinical intrafamilial and interfamilial variability remains incompletely understood [11].

Despite the well-established molecular basis of CHH, its clinical and genetic characteristics in the Mexican population remain poorly documented. Given its phenotypic variability and rarity, establishing a diagnosis can be challenging. Here, we describe eight Mexican patients with biallelic *RMRP* variants and features consistent with CHH, expanding the clinical and molecular spectrum of this condition.

## 2. Results

### 2.1. Clinical and Radiographic Features

Among the eight patients included in the study, four were male and four were female. At the time of evaluation, their ages ranged from 3 months to 12 years, with a mean age of 4.5 years.

Disproportionate short stature was observed in all patients (100%). Joint hypermobility was present in five patients (62.5%). Prenatal shortening of the long bones, short stature at birth, and femoral bowing were each observed in four patients (50.0%), whereas sparse hair was reported in three patients (37.5%) (Table 1).

**Figure 1:**
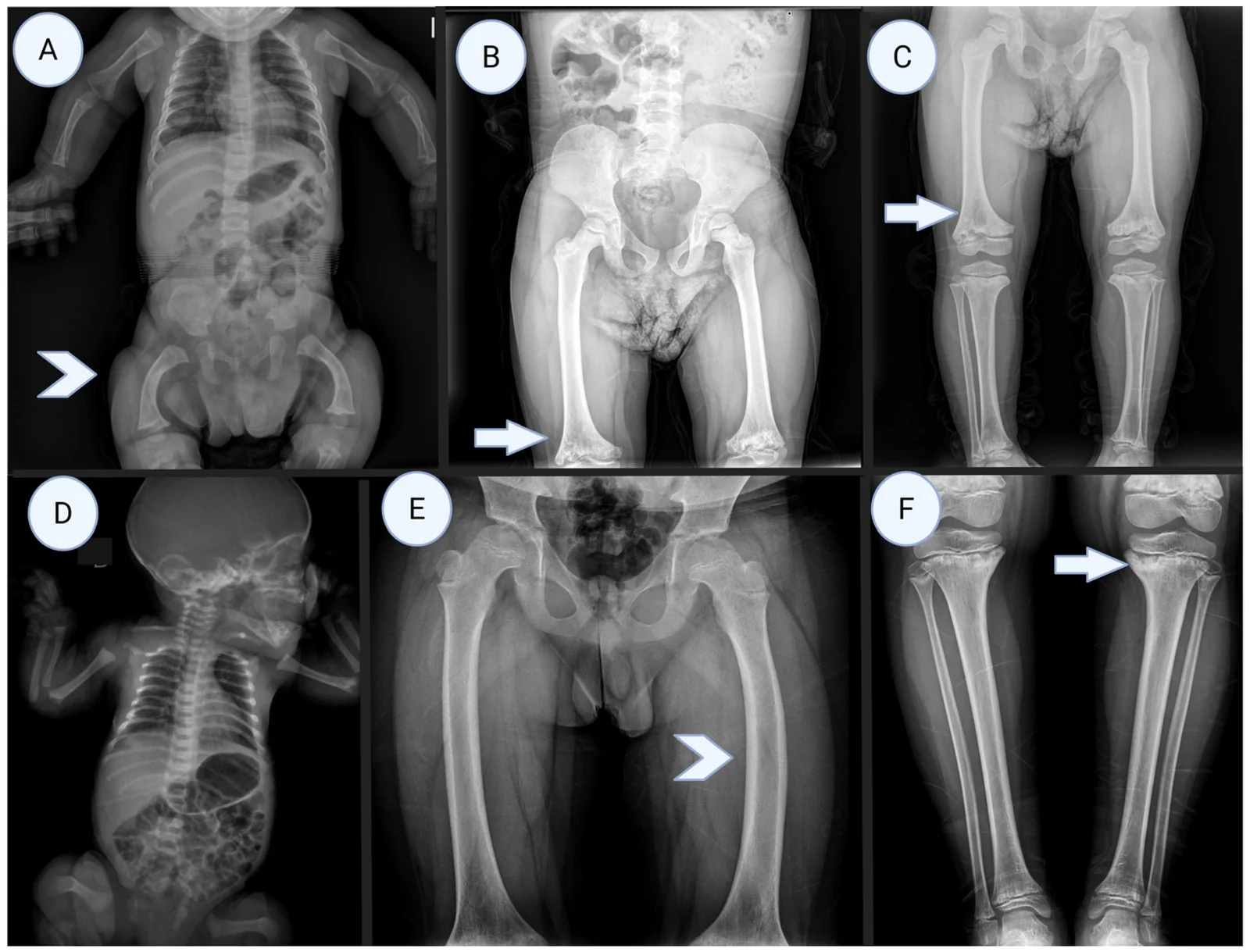
**Representative radiographic findings in patients with CHH.** (**A**–**F**), representative radiographs showing shortened long bones, femoral bowing (white arrowheads), and metaphyseal dysplasia. Flaring of the metaphyseal ends is indicated by white arrows in panels (**B**,**C**,**F**). (**A**), Patient 2; (**B**,**C**), Patient 3; (**D**), Patient 4; (**E**,**F**), Patient 6. Figure created with BioRender.

**Figure 2:**
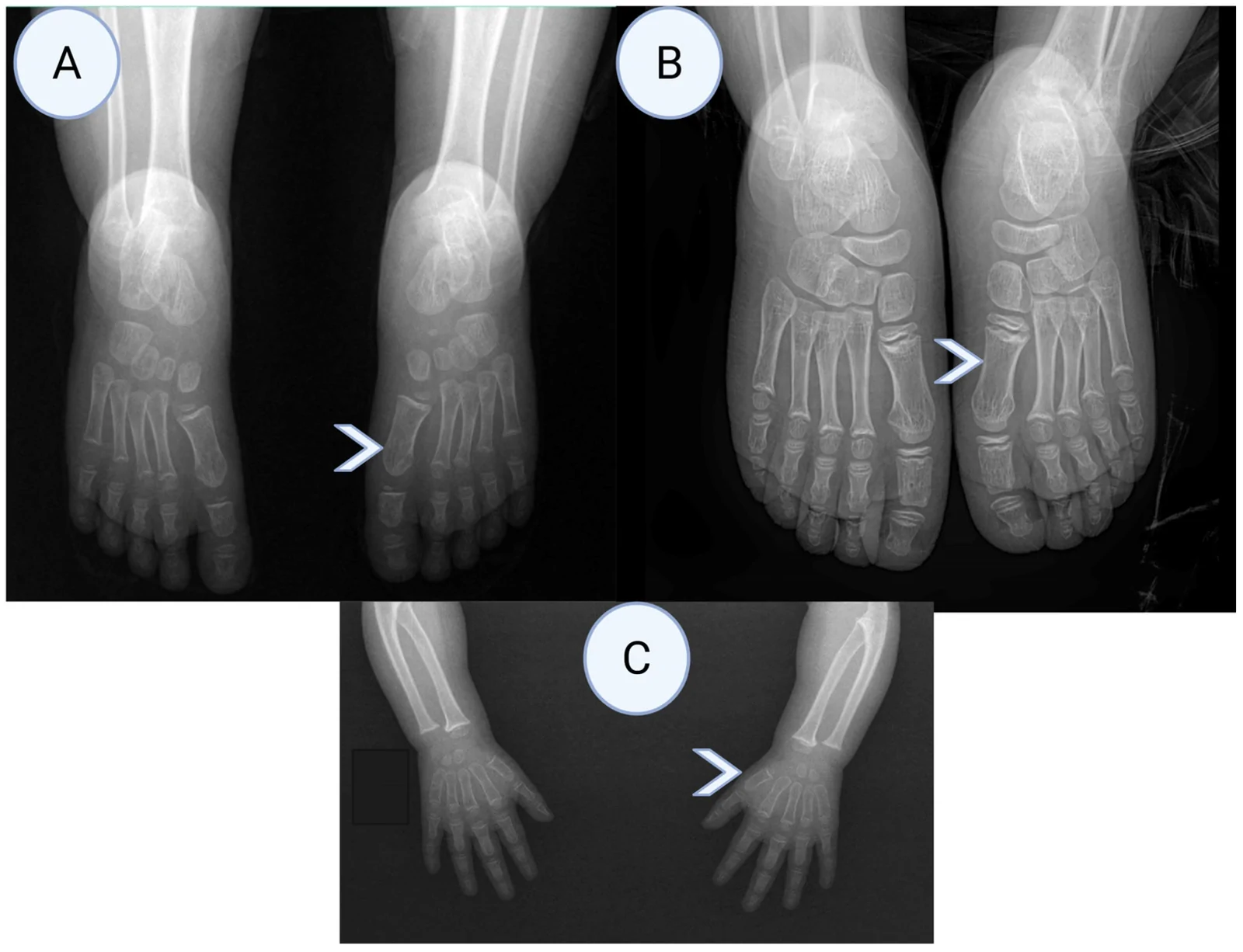
**Representative radiographs of short, cone-shaped phalanges in patients with CHH.** (**A**,**B**), short, cone-shaped phalanges and metatarsal bones (white arrowheads) in the feet of two patients. (**C**), short, cone-shaped phalanges and metacarpal bones (white arrowheads) in the hand of a patient. (**A**,**C**), Patient 5; (**B**), Patient 3. Figure created with BioRender.

Of the seven patients with available radiographic data, all had widened long bones and metaphyseal dysplasia (100%) (Figure 1). Short, cone-shaped phalanges were observed in six patients (85.7%) (Figure 2), and narrowing of the interpedicular distance was observed in three patients (42.9%).

Extraskeletal manifestations were identified in four patients. Patient 1 exhibited multiple features, including leiomyoma, impaired spermatogenesis, bronchiectasis, and lymphopenia. In contrast, patients 2 and 3 each had a single feature, namely Hirschsprung disease and anemia, respectively. Patient 8 also presented with immunodeficiency, which was detected at 3 months of age following newborn screening. Given the severity of her immunodeficiency, she subsequently underwent hematopoietic stem cell transplantation at 11 months of age, with her father serving as the donor (Table S1).

### 2.2. Genetic Diagnosis

Eight distinct *RMRP* variants were identified among the eight patients from seven unrelated families, comprising two homozygous genotypes in patients 2 and 5, whose parents were non-consanguineous, and six compound heterozygous genotypes (Table 2). Parental analysis confirmed that each parent carried one of the two variants identified in the affected offspring, consistent with the autosomal recessive inheritance of CHH. Neither parent displayed clinical features suggestive of CHH based on the clinical information collected.

The n.72A>G variant was the most common, accounting for 6 of 14 unrelated alleles (42.9%). Notably, the promoter deletion n.-39_-19del had not been previously reported and was identified in two affected siblings (Patients 6 and 7), in whom it occurred in trans with the likely pathogenic n.125C>T variant. The n.-39_-19del variant was absent from population databases and from available exome data from 177 unrelated Mexican individuals. According to the ClinGen *RMRP*-specific specifications (GN088, Version 1.3), the variant was classified as likely pathogenic based on its absence from population databases (PM2_Supporting), its occurrence in trans with the likely pathogenic n.125C>T variant in the two affected siblings (PM3_Moderate), its segregation in both affected siblings and absence in an unaffected sibling carrying only n.125C>T (PP1_Supporting), and the presence of disproportionate short stature and radiographic metaphyseal dysplasia in both affected siblings, consistent with the characteristic phenotype of CHH (PP4_Moderate) [13].

## 3. Discussion

### 3.1. Clinical Characteristics

We report eight patients with cartilage-hair hypoplasia from seven families. Although CHH is rare, it should be considered in the differential diagnosis of disproportionate short stature, particularly when characteristic skeletal and radiographic findings are present. Although several Mexican disease-specific cohorts have reported genetic causes of short stature, including achondroplasia and Noonan syndrome, their relative contribution to short stature at the population level remains unknown. Similarly, the frequency of CHH in Mexico has not been established because population-based epidemiological data are lacking. Our findings therefore contribute to the limited clinical and molecular characterization of CHH in the Mexican population.

In our cohort, prenatal findings suggestive of skeletal dysplasia were detected in 4 of 8 patients (50%), compared with 32 of 42 patients (76.2%) with available prenatal ultrasound data in the Finnish cohort of 108 patients [1]. This discrepancy should be interpreted cautiously, as prenatal data were available for only 42 Finnish patients. Variation in referral patterns, prenatal surveillance, and the operator-dependent nature of ultrasonographic assessment may have influenced detection rates. In addition, the Finnish cohort included multiple affected individuals from the same families, potentially resulting in more targeted surveillance in subsequent pregnancies. Thus, the observed difference in prenatal detection may reflect differences in prenatal assessment and ascertainment rather than in the prenatal skeletal phenotype of CHH.

Notably, CHH-related findings became evident during infancy in 62.5% of our patients (5/8), supporting the variable age at which the skeletal phenotype becomes clinically recognizable [12,14]. Severe growth restriction at birth has been hypothesized to be associated with early mortality in patients with CHH; however, this association has not been confirmed [4,12]. Instead, extraskeletal complications may contribute more substantially to long-term morbidity and survival. In our cohort, short stature at birth was documented in 50% of patients (4/8), three of whom also presented with extraskeletal complications. Given the young age of our patients (3 months to 12 years), close and continued clinical follow-up is essential to monitor complications and assess long-term outcomes.

In our cohort, widened long bones and metaphyseal dysplasia were the most consistently observed radiographic features, present in all patients (100%), followed by short, cone-shaped phalanges (85.7%), femoral bowing (50%), and narrowing of the interpedicular distance (42.9%). These findings are consistent with the characteristic skeletal phenotype of CHH [2,15]. However, its radiographic expression is age-dependent and may vary considerably among patients, with some characteristic features absent at certain stages [2,16,17]. Sparse hair is a well-recognized manifestation of CHH and has historically contributed to its clinical diagnosis; however, it may be absent in approximately half of affected individuals [1]. In our cohort, sparse hair was observed in 37.5% of patients (3/8).

CHH is associated with a broad spectrum of extraskeletal manifestations, some of which have been linked to more severe disease and poorer outcomes [4,5,18,19]. In our cohort, patient 8 was diagnosed with immunodeficiency at 3 months of age through newborn screening and subsequently underwent hematopoietic stem cell transplantation at 11 months of age. At the last follow-up, she had no history of recurrent infections. Nevertheless, immune abnormalities may remain clinically silent, supporting the value of screening even in asymptomatic patients with CHH [5]. Patient 1, who had lymphopenia, experienced recurrent infections during early infancy but remained asymptomatic at the most recent follow-up. Immunological screening was not performed in the remaining six patients because none had a history of recurrent infections. However, subclinical or undiagnosed immune dysfunction cannot be excluded, representing a limitation of our study.

Macrocytic anemia is a common manifestation of CHH, reported in approximately 73% of patients and often improving during childhood [18]. In our cohort, only patient 3 (12.5%) developed macrocytic anemia during infancy, with a favorable course. Hirschsprung disease was identified in patient 2, a recognized gastrointestinal feature of CHH [19]; despite its reported association with more severe disease, this patient has remained free of major complications. No malignancies were observed; however, given the young age of our cohort and the reported risk of lymphoma and skin cancer later in life, long-term surveillance remains warranted [5]. Other findings included impaired spermatogenesis and leiomyoma in patient 1. While impaired spermatogenesis is a recognized manifestation of CHH, an association with leiomyoma has not been established, and its occurrence may be incidental [20].

Overall, the clinical and extraskeletal manifestations observed in our cohort were consistent with the broad phenotypic spectrum previously reported in CHH. However, the small sample size does not allow us to determine whether specific phenotypic features distinguish Mexican patients from other populations.

### 3.2. Genetic Diagnosis

The genetic spectrum of CHH is broad and includes a wide range of *RMRP* variants. The recurrent n.72A>G allele is the most extensively studied and is strongly associated with the classical form of CHH described in Amish and Finnish populations. Functional studies have shown that this variant alters RNA processing, reducing cyclin B mRNA cleavage by approximately 40% and rRNA processing by 20% [11]. In our cohort, n.72A>G was the most common allele, identified in six of 14 unrelated alleles (42.9%), including one homozygous and four compound heterozygous patients. This proportion was lower than that reported among Finnish patients but similar to the approximately 48% reported among non-Finnish patients [2]. These findings do not indicate a molecular spectrum specific to the Mexican population.

Four of the five patients carrying n.72A>G presented with complex manifestations, including anemia, Hirschsprung disease, immunodeficiency, and impaired spermatogenesis. Although genotype–phenotype correlations have been proposed, findings from other populations and our cohort demonstrate substantial clinical variability among both homozygous and compound heterozygous individuals [12]. The causes of clinical variability in CHH remain unclear, particularly among patients carrying the same *RMRP* variants. Potential contributors include allelic combinations, additional genetic or coexisting genetic conditions, epigenetic factors, and variants in genes encoding components of the RNase MRP complex, particularly *POP1* and *NEPRO* [21]. To explore the potential contribution of *POP1*, we analyzed Patients 1–6 and did not identify any variants predicted to affect its expression or potentially modify the clinical presentation. Further studies are needed to investigate these and other factors that underlie the phenotypic variability.

Among the other pathogenic variants identified, n.147G>A has been reported predominantly in European patients. Functional studies suggest that this variant reduces cyclin B mRNA cleavage by approximately 5% and rRNA processing by 25% [11]. In our cohort, patient 1, who carried n.147G>A in combination with n.72A>G, exhibited several extraskeletal manifestations, including leiomyoma, impaired spermatogenesis, bronchiectasis, and lymphopenia. The n.6C>T variant, historically designated n.4C>T, has been reported in patients of English, German, and Spanish origin. Compound heterozygotes carrying this variant have been described with skeletal dysplasia and little or no hair hypoplasia, along with variable extraskeletal manifestations [11], consistent with the phenotype observed in patient 8.

Two additional variants, n.127C>T and n.125C>T, were classified as likely pathogenic. The n.127C>T variant has been reported in at least three individuals with CHH in LOVD (Leiden Open Variation Database), although detailed phenotypic information is unavailable [22]. In our cohort, n.127C>T occurred in combination with n.72A>G in patient 4, who, notably, was the only carrier of n.72A>G without reported extraskeletal manifestations.

The n.125C>T variant has been reported in at least one patient with CHH and malabsorption [9] and was identified in affected siblings in combination with the novel promoter deletion n.-39_-19del. Promoter variants are recognized causes of CHH, with numerous pathogenic or likely pathogenic indels, deletions, and duplications reported in ClinVar. Although most have been described in compound heterozygous states with an intragenic variant, at least three patients with biallelic promoter variants have been reported, including the compound heterozygous c.-4_-1dup/c.-21_-14trip and homozygous c.-19_-25dup and c.-13_-6dup [6]. Functional analysis of the homozygous c.-13_-6dup showed a severe reduction in *RMRP* transcript abundance to approximately 10% of control levels [6]. In our patients, the novel n.-39_-19del variant encompasses the *RMRP* TATA box (n.-32 to n.-24), a critical regulatory element for transcription initiation. Although its functional effect was not assessed, the deletion may impair promoter function and reduce *RMRP* expression.

The current ClinGen *RMRP* variant interpretation specifications (GN088, Version 1.3) do not formally assign PM1 to this variant, as the criterion is restricted to insertions or duplications located between the TATA box and the transcription start site. Nevertheless, the location of n.-39_-19del provides additional biologically relevant evidence supporting its interpretation. Together with its absence from population databases, confirmed occurrence in trans with the likely pathogenic n.125C>T variant, segregation with the phenotype in the affected siblings, and their characteristic skeletal phenotype, these findings support the classification of n.-39_-19del as likely pathogenic.

Two variants of uncertain significance (VUSs), n.128G>C and n.215C>T, were identified. The former was present in one of 14 unrelated alleles (7.1%), whereas the latter was found in the homozygous state in patient 5, whose parents reported no consanguinity, representing two of the 14 alleles (14.3%) analyzed. The n.128G>C variant has been reported in the homozygous state in patients with a primary immunodeficiency phenotype [10], whereas no previous association between n.215C>T and CHH has been reported. To further assess population frequency, 177 unrelated Mexican individuals were screened for the eight variants. All were absent except n.128G>C, which was detected in one heterozygous individual (1/354 alleles; 0.28%). Both VUSs showed low population frequencies and high conservation scores, providing supportive but insufficient evidence for pathogenicity. Functional studies are important to clarify their clinical significance [3].

Taken together, our findings expand the clinical and molecular spectrum of CHH in a Mexican cohort and highlight the need for population-based studies to better define its epidemiology in Mexico.

## 4. Materials and Methods

### 4.1. Subjects

Patients were recruited from two tertiary care hospitals in Guadalajara, Jalisco, Mexico, over a four-year period as part of the research protocol entitled “Analysis of the Clinical and Molecular Diagnosis of Twelve Skeletal Disorders in Mexican Patients,” conducted at the Instituto Mexicano del Seguro Social (IMSS). All participants underwent a comprehensive clinical evaluation by a medical geneticist, and CHH was suspected based on overall clinical and radiographic findings. Radiographs were independently evaluated by the medical geneticist responsible for the clinical assessment of each patient and by the medical geneticist compiling the data for this study. Both assessments focused on the skeletal findings relevant to the suspected diagnosis of CHH, including metaphyseal abnormalities, cone-shaped phalanges, and other characteristic radiographic features. No discrepancies were identified between the independent evaluations. Patients with disproportionate short stature and clinical and radiographic findings suggestive of CHH were selected for molecular analysis of the *RMRP* gene.

A total of eight patients from seven unrelated families were enrolled in the study. Written informed consent was provided by the parents or legal guardians of all minor patients, as well as by the parents who contributed blood samples for molecular analysis. Age-appropriate assent was obtained from the children when applicable. The study was conducted in accordance with the principles of the Declaration of Helsinki and was approved by the appropriate IMSS Research and Ethics Committees.

### 4.2. Molecular Analysis

#### 4.2.1. Genomic DNA Extraction

Peripheral blood samples (2–4 mL) were collected in EDTA-containing tubes. Genomic DNA was extracted from whole blood using the dodecyltrimethylammonium bromide–cetyltrimethylammonium bromide (DTAB–CTAB) method, as previously described [23].

#### 4.2.2. Polymerase Chain Reaction

The entire transcribed region of the *RMRP* gene was amplified by polymerase chain reaction (PCR) and subsequently analyzed by Sanger sequencing. Primers were designed using Oligo 6.0 software. The forward primer was 5′-AAACGGTTTTTTTAATCTCACGC-3′ and the reverse primer was 5′-ACTACTTGGGAGCCTGAGGTG-3′, generating a 450-bp amplicon. PCR was performed using a touchdown protocol consisting of three stages, with annealing temperatures of 63 °C, 62 °C, and 61 °C for 5, 5, and 25 cycles, respectively. During these stages, the denaturation times were 15, 15, and 5 s, and annealing times were 20, 17, and 17 s at the corresponding temperatures. Extension was carried out at 72 °C for 20 s in all cycles. Each 10 µL reaction contained 50–100 ng of genomic DNA, 1.5 mM MgCl_2_, 2.5 pmol of each primer, 1× PCR buffer, 5% DMSO, 0.1 U of recombinant Taq DNA polymerase (EP0404), and 0.204 mM of each dNTP. The PCR products were purified using 0.5 µL of ExoSAP-IT cleanup reagent (Applied Biosystems, Waltham, MA, USA), followed by incubation at 37 °C for 15 min and 80 °C for 15 min.

#### 4.2.3. Sanger Sequencing

Purified PCR products were subjected to bidirectional Sanger sequencing. Each reaction was carried out in a final volume of 10 µL containing 100 ng of DNA, 0.5 µL of Big Dye Terminator v.3.1 Ready Reaction Mix (Applied Biosystems, Waltham, MA, USA),1.5 µL of 5× sequencing buffer, and 2.5 pmol of primer. The thermal cycling conditions consisted of an initial denaturation at 96 °C for 4 min, followed by 25 cycles of denaturation at 96 °C for 10 s, annealing at 55 °C for 5 s, and extension at 60 °C for 2 min. Sequencing products were purified using columns containing Sephadex G-50 resin (Cytiva Life Sciences, Uppsala, Sweden) and analyzed by capillary electrophoresis on a SeqStudio Genetic Analyzer (Applied Biosystems).

Electropherograms were reviewed manually and analyzed using SnackVar software, version 2.4.3 [24]. Sequence variants were identified by comparison with the reference *RMRP* sequence NR_003051.4 and described according to the Human Genome Variation Society (HGVS) recommendations [25]. The clinical significance of identified variants was assessed based on public databases and published reports. The novel *RMRP* n.-39_-19del variant was classified according to the ClinGen *RMRP*-specific specifications (GN088, version 1.3), incorporating applicable ClinGen SVI recommendations and guidance for the interpretation of non-coding variants.

## 5. Conclusions

This study expands the clinical and molecular spectrum of CHH in a Mexican cohort by documenting its skeletal and extraskeletal manifestations and identifying a previously unreported RMRP promoter deletion, n.-39_-19del, which was classified as likely pathogenic.

The recurrent n.72A>G variant was the most common pathogenic allele identified in our study. Clinical variability was observed among patients carrying the same *RMRP* variants, suggesting that additional genetic and epigenetic factors may contribute to phenotypic differences, although these factors were not specifically investigated.

The findings highlight the challenges of interpreting non-coding *RMRP* variants and support the contribution of promoter alterations to CHH. Functional studies are needed to further clarify the clinical significance of n.-39_-19del and the two VUSs identified in this study, including quantitative assessment of *RMRP* transcript levels and potential effects on RNA structure. Overall, these results support the integration of clinical, radiographic, and molecular assessment for the diagnosis and characterization of CHH, particularly in populations with limited molecular data.

## Figures and Tables

**Table 1:** Frequency of clinical and radiographic features in patients with cartilage-hair hypoplasia.

Features	*n/N*	%
Prenatal features		
Shortened long bones on prenatal ultrasound	4/8	50.0
Neonatal features		
Short stature at birth	4/8	50.0
Infancy and childhood features		
Disproportionate short stature	8/8	100.0
Joint hypermobility	5/8	62.5
Femoral bowing	4/8	50.0
Sparse hair	3/8	37.5
Radiographic features		
Widened long bones	7/7	100.0
Metaphyseal dysplasia	7/7	100.0
Narrowing of the interpedicular distance	3/7	42.9
Short, cone-shaped phalanges	6/7	85.7

Note: *n*: number affected; *N* = number evaluated.

**Table 2:** *RMRP* variants identified in patients with cartilage-hair hypoplasia.

Patient	Maternal Allele	Paternal Allele
1	n.147G>A (rs753874439)	n.72A>G (rs199476103)
2	n.72A>G	n.72A>G
3	n.72A>G	n.128G>C (rs74810894)
4	n.72A>G	n.127C>T (rs1303461251)
5	n.215C>T (rs772665785)	n.215C>T
6 and 7 *	n.125C>T (rs938089991)	^†^ n.-39_-19del
8	n.72A>G	n.6C>T (rs772443941)

* Patients 6 and 7 are siblings. ^†^ The novel n.-39_-19del variant was classified as likely pathogenic according to the ClinGen *RMRP*-specific specifications (GN088) based on PM2_Supporting, PM3_Moderate, and PP1_Supporting.

## Data Availability

The original contributions presented in this study are included in the article and Supplementary Materials. Further inquiries can be directed to the corresponding author.

## Supplementary Materials

The following supporting information can be downloaded at: https://www.mdpi.com/article/10.3390/ijms27198815/s1.
